# Optimal age of the donor graft tissue in relation to cultured pearl phenotypes in the mollusc, *Pinctada margaritifera*

**DOI:** 10.1371/journal.pone.0198505

**Published:** 2018-06-18

**Authors:** Carole Blay, Serge Planes, Chin-Long Ky

**Affiliations:** 1 Ifremer, UMR 241, EIO, Labex Corail, Centre du Pacifique, Taravao, Tahiti, Polynésie Française; 2 PSL Research University, EPHE-UPVD-CNRS, USR 3278 CRIOBE, Labex Corail, Université de Perpignan, Perpignan, France; Laboratoire de Biologie du Développement de Villefranche-sur-Mer, FRANCE

## Abstract

Ageing is defined as the progressive decline in tissue and organ functions over time. This study aims to evaluate the ageing effect on cultured pearl quality phenotypes (including size and quality traits) in the graft-recipient animal model: *Pinctada margaritifera*. For this, eight uniform grafting experiments were designed using two hatchery-produced pearl oyster families as donors, which were followed through time, between 7 and 30 months in age. For each age category, 20 donors were studied for each culture site giving a total of 2400 grafted oysters. Several phenotypic measurements were made: 1) donor family growth performance from shell size records, 2) pearl size and corresponding quality traits, and 3) expression of some genes related to biomineralization processes on both the mantle graft and on pearl sac tissues. Results showed that: 1) donor age has an impact on pearl size, with grafts coming from the youngest donors yielding the biggest pearls; and 2) grafts from donors between 12 and 18 months in age produced pearls of the highest quality (grade and surface quality), a result supported by an analysis where the level of expression for a panel of genes associated with biomineralization was greatest in donors within the 12 to 18 months age group. These results indicate that donors aged between 12 and 18 months have high potential for biomineralisation and nacre deposition, and likely produce larger and higher quality cultured pearls than older donors.

## Introduction

The process of ageing affects living organisms, from single cell yeasts to multi cellular animals and plants. Most evolutionary biologists define ageing as the age-dependent or age-progressive decline in tissue function over time [1–10]. In a graft context, it is tempting to speculate that donor age determines graft/ scion quality and further, the long-term function after transplantation. Cellular dynamism and more specifically cell age has been studied through numerous graft models. In human models, there is an upper age limit for the donor in many organ transplant centers. For example, some researchers reported that older livers had a higher rate of primary non-function, prolonged graft function recovery, and an increase in graft loss or mortality [11–15]. In the plant kingdom, the relative effect of age on tree metabolism revealed age-mediated controls for tree growth, which are particularly important in the first years of its life [16,17]. Another study examined biological and environmental factors that control root dynamics and function through the effect of root ageing on grapevines [18].

Since the development of artificial pearl cultured techniques in the early 1900’s, the production of nucleated culture marine pearls has become a significant industry throughout Southeast Asia, northern Australia and Pacific Island nations [19]. The marine pearl industry is primarily based on the culture of pearl oysters from the genus *Pinctada* spp or *Pteria* spp (Family: Pteriidae) [20], with an estimated annual global value of over US$397million in 2013 [21]. The black-lipped pearl oyster *Pinctada margaritifera*, mainly from French Polynesia, with minor productions in the Cook Islands, Fiji, the Marshall Islands and Micronesia, is valued for its ability to produce high quality colored pearls. From a genetic point of view, grafting associates two tissues from different individuals, the recipient and donor (two distinct genomes) that create a genetic chimera with each genome maintaining its own genetic identity throughout the grafted organism [22]. To initiate pearl formation in our model *P*. *margaritifera*, the gonad of a “recipient” oyster is surgically implanted with a spherical shell bead nucleus (made from the shell of a freshwater mussel from the Mississippi River) alongside a small graft (~4mm^2^) of mantle tissue cut from a donor oyster. This small piece of donor mantle tissue is called saibo. The saibo grows around the nucleus and becomes a “pearl sac”, which secretes successive nacre layers on the nucleus. This process results in the formation of a cultured pearl in about 15–20 months. As only small pieces of saibo are required for grafts, a single donor oyster can provide material for multiple recipients. Once a pearl has been harvested, it is then classified based on several factors that, in combination, will determine its quality and therefore its market value [19].

*Pinctada margaritifera* cultured pearls are still produced using wild populations due to the abundance of natural oyster resources and, until recently, the challenges associated with controlling the reproduction and early life stages in culture. Rearing of the species over its entire life cycle, including artificial breeding, became possible through the domestication of *P*. *margaritifera*. This advance has allowed oyster age to be controlled, in contrast to current Polynesian pearl aquaculture methods which rely on wild spat collection where collected individuals are of undetermined age. To date, studies that examined pearl quality traits mainly focused on the genetics of the donor oyster. The influence of the donor on pearl quality traits has been definitively demonstrated using reciprocal xenografts between *P*. *maxima* and *P*. *margaritifera* in which donors were found to have a significant effect on both color and surface complexion [23]. Studies equally demonstrated a donor [24] or family [25] effect on pearl quality traits (color, darkness, surface defect, lustre and grade). The influence of culture site (Island) on pearl quality traits has also been reported [26–29]. A recent study evaluated the effect of donor shell size at a specific age on pearl quality traits [30] and found that the size of the donor shell does not impact the size or quality of the pearl. Despite existing knowledge about donor and recipient roles on pearl quality traits, only one previous study examined the effects that the age of donor oyster might have on pearl grade, lustre, surface defect and color. The study found that pearls produced from 2 year old donors were significantly better quality than those produced by 5 year old donors [31]. No study has examined the impact of using a younger donor oyster (< 2 years old) and the age effect on cultured pearl size.

This study investigated the age effect (independent from shell size) of hatchery-produced donor *P*. *margaritifera*, on the culture pearl phenotypes and on the potential for biomineralization. To do this, we conducted grafting experiments using donors from a single biparental family at 12, 18, 24 and 30 months of age, and assessed resulting pearl quality from each cohort. To determine whether our results were reproducible (and to assess even younger donors), we conducted a second series of experiments in a separate location, in which donors from a second unrelated family were used as grafts at 7, 12, 18 and 24 months of age. Several phenotypic measurements were made: 1) the shell biometric growth parameters of the donor families, 2) the cultured pearl size and quality traits, and 3) the expression of eight gene encoding proteins involved in the biomineralization process in both the mantle graft of the donor and in the final pearl sac tissue. In the end, this study contributes to the optimization of the graft process by identifying the ideal age for the donor oyster which then improves pearl quality traits in hatchery pearl culture systems.

## Materials and methods

### Ethics statement

The pearl oysters used in the current study are marine-cultured animals, and all of the experiments on pearl oysters were conducted following the institutional and national guidelines. The authority who issued the permission for pearl oysters transfers is: "Direction des Ressources Marines et Minières". No endangered or protected species is involved in the present study, and no specific permission is required for the location of the culture experiment, as it is in public maritime area.

### Grow-out site locations: Rangiroa atoll and Mangareva Island

Rangiroa atoll is located in the north-western part of the Tuamotu Archipelago (15°07’S, 147°38’W), about 350 km northeast of Tahiti. The atoll has a flattened elliptic shape, is 80 km in length and ranges from 5 to 32 km in width. The lagoon surface is 1446 km^2^. The site of culture is located at Avaturu village (Gauguin’s pearl farm).

Mangareva Island is located in the Gambier Archipelago (23°09′S, 134°58′W), about 1590 km southeast of Tahiti. The island surface area is 27 km^2^. The island is the central and largest of the archipelago and has a large lagoon 24 km in diameter containing reefs. The culture site was located at Regahiga Pearl Farm.

### Animal sources

Two bi-parental *P*. *margaritifera* families were produced at the Ifremer hatchery facilities (Vairao—Tahiti, French Polynesia), using wild female and male broodstock from Rangiroa Island (Tuamutu Archipelago, French Polynesia) for F_RGI family, and Mangareva Island (Gambier Archipelago, French Polynesia) for F_GMR family. Spawning was induced by thermal shock [32]. Artificial breeding, larval rearing and oyster culture procedures were conducted using the protocol developed by Ky et al. [25]. After approximately 45 days of age, the seed oysters were transferred to the nursery and reared in raceways of 90 × 20 × 20 cm (corresponding to a volume of 30 liters). Unfiltered seawater was added with a suspension of algae produced in outdoor tanks at a renewal rate of 100 L h-1. The juveniles were detached once they reached an average size of 3.0 ± 0.8 mm (dorso-ventral measurements) and were pierced and tied together onto a CTN (Cord Technical Nakasai) rearing system, where they were left until their transfer at 6 months old. The CTN involves drilling a small hole through the base of the shell in the dorsal-posterior region. This process does not affect living tissue. The CTN were protected using plastic mesh to prevent predation in the lagoon.

CTN from the two families, used as donors, were randomly selected and transferred (at six months old) by plane to Rangiroa atoll for F_RGI family and Mangareva Island for F_GMR family to allow the oysters to adapt (1 month) and grow in local environmental conditions before they were randomly selected for the grafting procedure. These pearl oysters (attached to CTN) were regularly cleaned by high pressure sea water spray

### Experimental design and grafting procedure

Donors from the F_RGI family were used in grafting experiments atthe Rangiroa atoll culture site at 12, 18, 24 and 30 months old (every 6 months). Twenty donors were randomly selected each time. F_GMR family was used in grafting experiments at Mangareva island culture site at 7, 12, 18 and 24 months old. Twenty donors were also randomly selected each time. All grafts were performed under standard production conditions by a single expert from each pearl farm, Gauguin’s pearl Farm (Rangiroa) and the Regahiga Pearl Farm (Gambier), to minimize the grafter effect on pearl quality phenotypes [33]. The recipient oysters were sourced from a local natural spat collection at each culture site. They were selected based on visible health status (colour of the visceral mass and gills), shell appearance (black colour without any damage, and with visible concentric growth lines) and muscle resistance when the shells were pried open. A total of 8 different grafting experiments were performed with all 80 donors for F_RGI and F_GMR families. These donors were used to perform 1400 and 1000 grafts, respectively (Table 1). All recipient oysters were seeded using 2.4BU nucleus size in Rangiroa and 1.8BU nucleus size in Mangareva. A larger number of grafts were performed in Rangiroa because the increased size of the donors enabled more pieces of saibo to be cut from the mantle. At 45 days post-graft, recipient oysters were checked to estimate nucleus retention and oyster mortality rates as described in [34]. After this check, recipient oysters that had retained their nuclei were fixed to chaplets after removing the net retention bags. Each chaplet was labelled according to the corresponding donor oyster for traceability. Furthermore, pearl oysters were regularly cleaned by high sea water spray every six months of culture at both culture sites, in order to remove biofouling (epibiota), which can hinder healthy oyster growth and pearl production.

**Table 1:** Summary of experimental design with location, experiment name and age of donor description. *Graft*: date of grafted procedure, number of saibo per graft (20 donor oysters were used for each experiment), total number of grafted oysters. *Harvest*: date of harvest (18 months post grafting), percentage of grafted oysters that produced a pearl and number of pearls and keshi. *Sample*: Number of graft tissues at graft time and number of pearl sac tissues at harvest time.

			Graft			Harvest				Sample	
Location	Experiment name	Age of donor (month)	Date	Number of saibo per donor	grafted oyster	Date	% Pearls	Pearls	Keshi	Graft tissue	Pearl sac tissue
Rangiroa	RGI12	12	sept-13	10	200	mar-15	31	62	10	19	20
	RGI18	18	mar-14	20	400	sept-15	23	93	8	20	20
	RGI24	24	sept-14	20	400	mar-16	29	115	12	20	19
	RGI30	30	mar-15	20	400	sept-16	33	130	4	20	19
Gambier	GMR7	7	oct-13	10	200	apr-15	63	125	10	0	19
	GMR12	12	mar-14	10	200	sept-15	44	88	10	18	0
	GMR18	18	sept-14	10	200	mar-16	80	159	3	19	19
	GMR24	24	mar-15	20	400	sept-16	79	314	8	20	19

### Cultured pearl quality phenotypes

Pearl phenotype categories were recorded to characterize the quality of the pearl [25]:

- Shape was characterized in two ways: the presence / absence of circle/s (shown by regular streaks or concave rings, whatever the shape category) and the shape category (“b” for baroque and semi baroque, “o” for oval and drop, “r” for round and semi round pearls).
- Color was evaluated through the darkness level (high, medium and low) and the visually perceived color category, which is conferred by bodycolour pigments and secondary colors (overtone). 5 major color categories were detected including green, grey, peacock, yellow and one named “other” including white, blue and aubergine pearls.
- Cultured pearl grade was determined for each sample according to the official A–D Tahitian classification (Journal Officiel 2001 n° 30, 26 July 2001) from the most to least valuable quality: A, B, C, D and Rejects (*rebuts*).
- Finally, surface defects and lustre (components of cultured pearl grade) were determined separately so that they could be studied independently. To ensure homogeneity in parameter assessment, all evaluations were made visually by the same operators.

### Biomineralisation gene expression phenotype

In order to assess expression levels of known biomineralisation genes in donor tissue of different ages, we sampled graft tissues (3 to 5 pieces per donor) during the graft operation and pearl sac tissues during the harvest. In order to minimize the mixture of recipient tissues, the pearl sacs were excised from host oysters by removing the outer layers with a surgical blade until a thin (< 0.5 mm) layer of tissue surrounding the pearls remained, and immediately transferred and preserved into 2.0 ml tubes with RNAlater”. Samples were preserved in RNAlater and stored at –80°C for subsequent RNA extraction to evaluate relative gene expression of aragonite-related genes (*Pif-177*, *MSI60*, *Perline*), calcite-related genes (*Aspein*, *Shematrin5*, *Prismalin*, *(for graft and pearl sac tissues) and Shematrin9* (only for pearl sac tissues)) and one gene implicated in both layers (*Nacrein*) (S1 Table). Two genes were used as housekeeping genes chosen based on their ubiquitous and constitutive expression pattern in *P*. *margaritifera* tissue: SAGE (SAGES: AGCCTAGTGTGGGGGTTGG/ SAGER: ACAGCGATGTACCCATTTCC) (called REF in [35] and GAPDH (GAPDHS: AGGCTTGATGACCACTGTCC/ GAPDHR: AGCCATTCCCGTCAACTTC) [36]. The relative stability of the GAPDH and SAGE combination was confirmed using NormFinder (Stability value for best combination) (Results in S1 Appendix).

After removing the RNAlater by pipetting and absorption, total cellular RNA was extracted from either the individual graft tissue or pearl sac samples, using TRIzol reagent (Life Technologies) according to the manufacturer’s recommendations. RNA was quantified using a NanoDrop ND-1000 spectrophotometer (NanoDrop Technologies, Inc.) and the quality of the RNA was checked to exclude degradation using an agilent 2100 bioanalyser. The RIN values were between 6.50 and 7.40 corresponding to a sufficient quality for quantitative real-time PCR analysis. Total RNA for each individual was then treated with DNAse I using a DNA-free Kit (Ambion). First strand cDNA was synthesized from 500 ng total RNA using the Transcriptor First Strand cDNA Synthesis Kit (Roche) and a mix of poly (dT) and random hexamer primers. Real-Time PCR amplifications were carried out on a Roche Light Cycler 480. A no-RT control was screened by qPCR using a housekeeper gene to ensure there was no DNA contamination. The amplification reaction contained 5 μL LC 480 SYBR Green I Master Mix (Roche), 4 μL cDNA template, and 1 μL of primer (1μM), in a final volume of 10 μL. Each run included a positive cDNA and a blank control for each primer pair. The run protocol was as follows: initial denaturation at 95°C for 10 min followed by 40 cycles of denaturation at 95°C for 30 s, annealing at 60°C for 30 s and extension at 72°C for 60 s. Lastly, the amplicon melting temperature curve was analyzed using a melting curve program: 45–95°C with a heating rate of 0.1°C s^-1^ and continuous fluorescence measurement. All measurements were made in duplicate and all analyses were based on the Ct values of the PCR products. We allowed a difference of less than 0.5 ct between our two replicates. If the difference is superior to 0.5 ct, the qPCR reaction was repeated, and the sample was removed if congruent ct values were once again not obtained.

The relative expression ratio (R) of a target gene was calculated based on E and the CP deviation of an unknown sample versus a “control”, and expressed in comparison to a reference gene as follows: R = E_(target)_^ΔCt target (control—sample)^ / E_(ref)_^ΔCt ref (control—sample)^ [37]. Here, the control represents the mean of the values obtained for the tested gene [38]. PCR efficiency (E) was estimated for each primer pair by determining the slopes of standard curves obtained from a serial dilution analysis of a cDNA to ensure that E ranged from 90 to 110% (S1 Table). A total of 136 graft tissues and 135 pearl sac samples were used for the analyses (last two columns in Table 1). The graft from GMR7 and pearl sac sample from the GMR12 experiment were not sampled due to technical problems.

### Measurements of shell biometric parameters and pearl size

Prior to the grafting operation, shell height, width and thickness of the 200 donor oysters were measured using Vernier calipers [28]. At 18 months post-graft (for each experiment and location), the cultured pearls were harvested and placed into a compartmented box that allowed traceability between sample pearls, the donor oyster and corresponding experiments. Once harvested, cultured pearls were cleaned by ultrasonication in soapy water with a LEO 801 laboratory cleaner (2-L capacity, 80 W, 46 kHz) and then rinsed in distilled water. The size of the cultured pearls was assessed by measuring nacre thickness and weight [27]. Pearl thickness was measured using a digital micrometer and nacre thickness = [(cultured pearl average diameter)—(nucleus diameter)]/2. The diameter of non-round pearls was taken as the averageof measurements from the thinnest and thickest points.

### Statistics

The normality of the data distribution and homogeneity of variance were tested for pearl size, donor oyster biometric parameters and relative gene expression ratio using the Shapiro-Wilk test and Bartlett’s test. When necessary, transformations were used to adjust data to the distribution (logarithm or square roots).

Group donor age was treated as a fixed variable. Firstly, an ANOVA test was performed to test age of donor effect on donor shell biometric parameters, cultured pearl weight, thickness, graft and pearl sac relative gene expression ratio. If the overall test was significant, a Dunn procedure with a Bonferroni correction for multiple tests was performed among all pairs of age groups. Qualitative classes based on cultured pearl surface defects, lustre, grade, darkness and circles were re-encoded to give quantitative scores that would enable the mean value of age group to be obtained for each criterion, thus allowing them to be ranked. Scores from 0 to 4 were attributed to the different classes from the least to the most valuable (with grade, surface defects, darkness and lustre). For each criterion, Kruskall-Wallis tests were then applied to compare the age and donor groups. For the cultured pearl “color categories” and shape categories, donors and times of harvest effect were compared using χ^2^ tests.

In all cases, the differences were considered statistically significant when *p* values were lower than 0.05. Statistical analyses were performed using R software (version 3.2.1)[39].

## Results

A total of 1086 pearls from the 8 experimental grafts were analyzed (1086 pearls from 2400 grafted oysters). We studied the impact of the age of donor oyster on the family shell growth, pearl size, pearl quality traits and relative gene expression in the graft and pearl sac tissue.

### Age effect on pearl size

**Fig 1:**
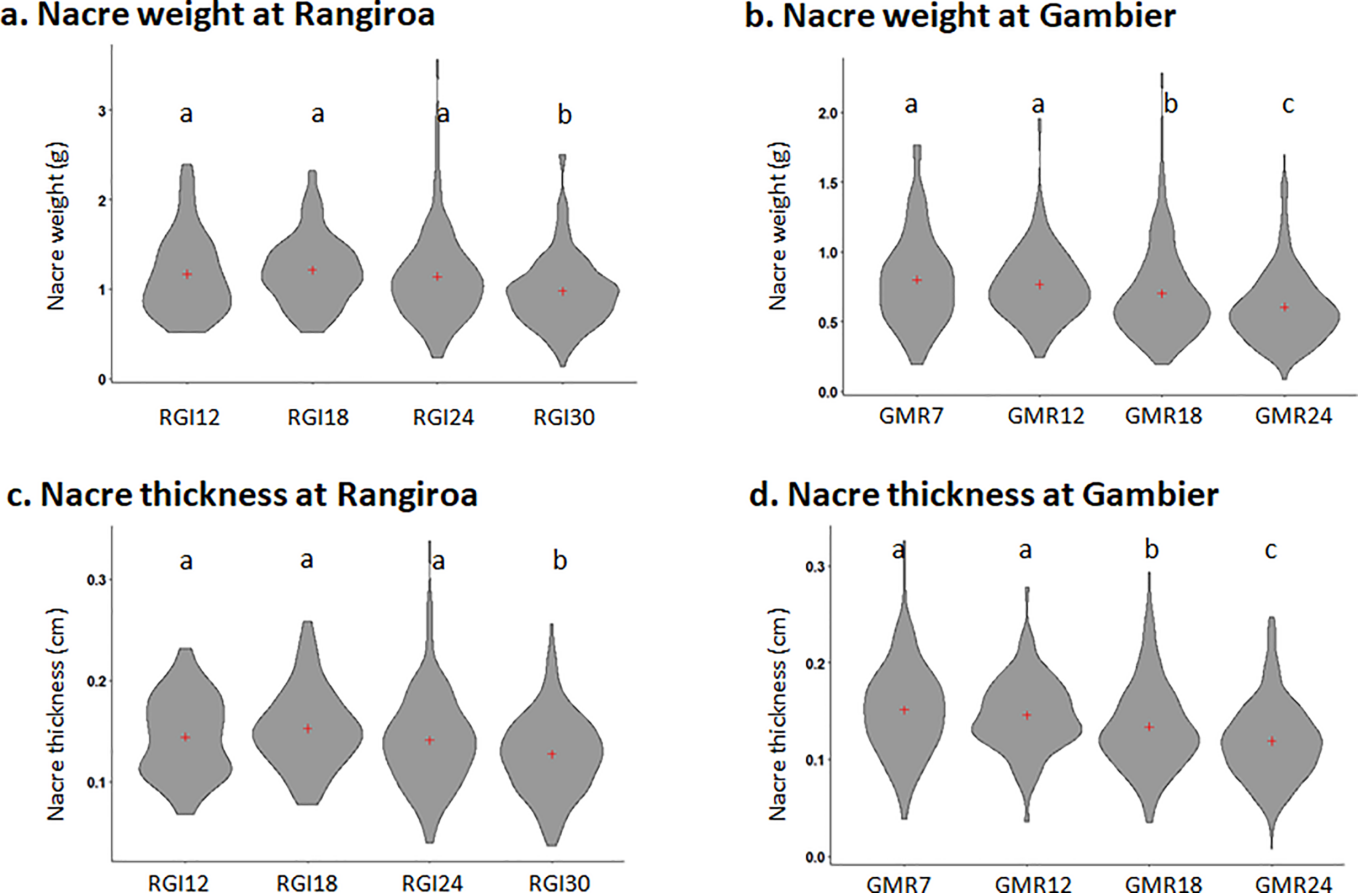
Pearl size harvested for the two sites across four age classes. Pearl nacre weight pearls in Rangiroa (a) and Gambier (b) and mean pearl nacre thickness in Rangiroa (c) and Gambier (d) measured across four age classes. “+” Cross represent the mean in the violin plot. Letter indicates significant difference between the age group (*p* < 0.05). Datas available on S2 Appendix.

Results of pearl size in Rangiroa and Gambier for each donor age category are illustrated in Fig 1. A highly significant age effect was recorded for nacre thickness and weight in Rangiroa and Gambier (p < 0.0001).

In Rangiroa, pearls from experiments RGI12, RGI18 and RGI24 were significantly thicker (p = 0.05, p < 0.0001 and p = 0.05, respectively) and heavier (p = 0.03, p = 0.0005 and p = 0.02, respectively) than pearls from experiment RGI30. Pearls from experiment RGI18 showed the greatest average nacre thickness (1.52 ± 0.39 mm) and weight (1.21 ± 0.38 g) and experiment RGI30 showed the thinnest (1.27 ± 0.39 mm) and lightest (0.98 ± 0.39 g), representing a difference of 17% nacre deposit and 23% nacre weight.

In Gambier, pearls from experiments GMR7, GMR12 were significantly thicker (p = 0.003 and p = 0.03, respectively) and heavier (p = 0.01 and p = 0.04, respectively) than pearls from experiments GMR18 and GMR24 (p < 0.0001). Moreover pearls from GMR18 were significantly thicker and heavier than pearls from GMR24 (p = 0.005 and p = 0.01, respectively). Pearls from experiment GMR7 showed the greatest average nacre thickness (1.51 ± 0.47) and weight (0.80 ± 0.33) and GMR24 showed the thinnest (1.19 ± 0.40 mm) and lightest (0.60 ± 0.26) pearls corresponding to a difference of 21% nacre deposit and 25% nacre weight.

### Age effect on pearl quality phenotypes

An age effect of high significance was detected for a number of cultured pearl surface defects in Gambier and Rangiroa (*p* = 0.01 and *p* = 0.001, respectively). Cultured pearls from GMR24 and RGI24 presented the “best” surface quality, with 40% and 51%, respectively of cultured pearls having less than 5 defects against GMR7 (24%) and RGI30 (22%). A large proportion of cultured pearls from GMR12 (48%) and RGI30 (48%) had up to 10 defects.

**Fig 2:**
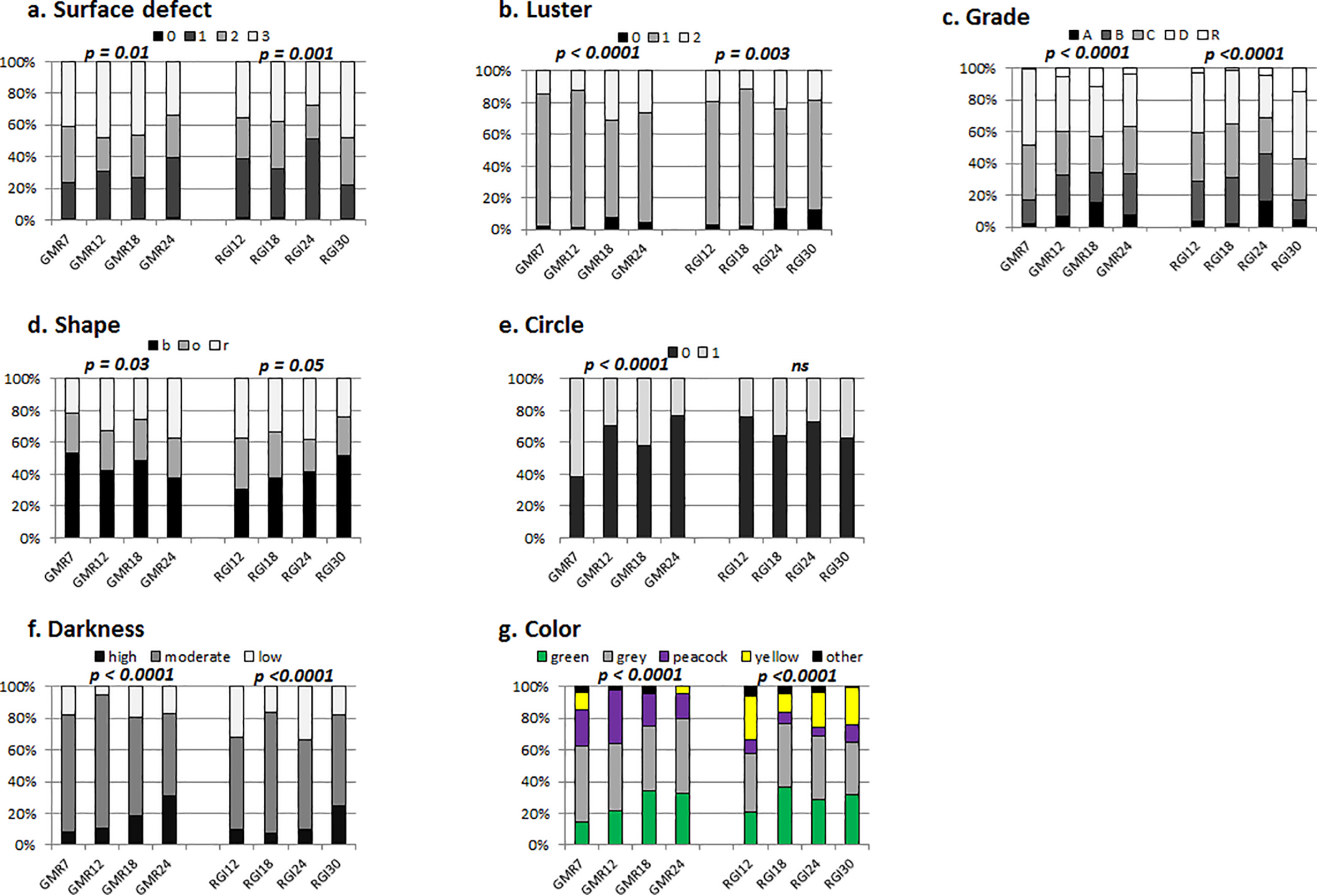
Cultured pearl quality traits from the experimental graft distribution. Percentage of cultured pearls for each experiment in Gambier (GMR) and Rangiroa (RGI) for different donors in different age groups (7, 12, 18, 24 and 30 months old) and the p-value in Gambier and Rangiroa with the following variables: a. surface defect classes (“0” = 0 defects, “1” = 1–5 defects, “2” = 6–10, and “3” = >10 defects), b. luster levels (“0” = absence of luster, “1” = moderate luster, and “2” = high luster), c. classification grade (“A” to “D” and Rejects), d. shape categories (“b” for baroque and semi baroque, “o” for oval and drop,” r” for round and semi round pearls), e. pearl circles (“0” = absence and “1” = presence), f. darkness level (low, moderate and high darkness) and g. visual color categories (“green”, “grey”, “peacock”, “yellow” and “other”, corresponding to white, blue and aubergine pearls). Datas available on S2 Appendix.

A significant age effect was recorded for “lustre” in Gambier and Rangiroa (*p* < 0.0001 and *p* = 0.003, respectively). Cultured pearls from GMR18 and RGI24 recorded the highest level of lustre pearl, but also the same amount of pearl without any lustre (Fig 2A).

Data analysis showed a significant age effect on cultured pearl grade (*p* < 0.0001). The pearls from GMR7 and RGI30 recorded the highest level of D-R pearls with a proportion of 49% and 57%, respectively, while GMR18 and RGI24 recorded the highest level of A-B pearls with 35% and 46%, respectively against 17% for GMR7 and 17% for RGI30. Cultured pearl quality trait distributions from each experimental graft are shown in Fig 2.

A low significant age effect was detected for shape categories in Rangiroa (p = 0.05) and Gambier (p = 0.03). No significant difference was recorded between the four different donor age experiments in Rangiroa for the absence or presence of circles, but a significant effect was recorded in Gambier (*p* < 0.0001). Pearls from GMR7 experiment were more circled than in other experiments with 62% of circled pearls compared to 31% of circled pearls, on average, in other experiments.

A significant age effect was recorded for darkness level: *p* < 0.0001. In Gambier, experiment GMR24 produced darker pearls where 31% of pearls were at the high darkness level, in contrast to only 9% recorded for GMR7 and GMR12. In Rangiroa, the darkest cultured pearls were found in experiment RGI30 which had 25% of the high darkness level whereas experiments RGI12, RGI18 and RGI24 produced around 9% of the high level of darkness.

A significant age effect was recorded for “color categories” (*p* < 0.0001). The different color proportions produced by the different experiments are illustrated in Fig 2.

### Age effect on biomineralisation process

**Fig 3:**
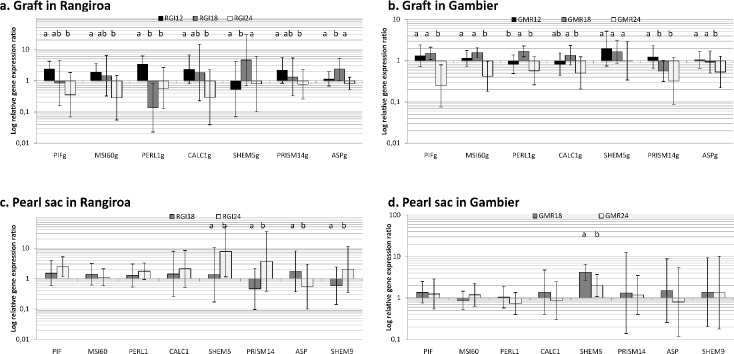
Relative gene expression ratio of biomineralisation genes in *P*. *margaritifera*. Relative expression of 7 genes in the saibo at Rangiroa (a) and Gambier (b) for donors aged 12, 18 and 24 months (dark to light grey). Relative expression of 8 biomineralization genes in the pearl sac in Rangiroa(c) and Gambier (d) for donors aged 18 and 24 months (dark and light grey, respectively). Y axes are in logarithmic scale. Error bars correspond to standard deviations. Statistical differences between the age groups are indicated by letters (*p* < 0.05). Datas available on S2 Appendix.

Relative gene expression for the panel of protein coding genes implicated in calcite and aragonite layers at Rangiroa and Gambier in the graft and pearl sac tissue are illustrated in Fig 3.

Concerning the graft tissue, the relative gene expression ratio for the seven candidates varied at Rangiroa and were significantly different among the three donor ages (12, 18 and 24 months). PIF, MSI60, PERL1 and CALC1 relative gene expression ratio were significantly higher for the 12 month old donors (2.40, 1.94, 3.51 and 2.24, respectively), compared to the 24 month old donors which had a relative gene expression ratio inferior to 0.6 for the four genes (*p* < 0.0001).

At Gambier, the relative expressions of 7 genes that encode for the biomineralization matrix proteins in the graft were significantly different between the donor age categories (p < 0.0001). PIF177, MSI60, PERL1, CALC1, ASP and PRISM14 relative gene expression ratio were significantly higher for GMR18 compared with GMR24 which had a relative gene expression ratio inferior to 0.6 for all genes (*p* < 0.0001).

In Rangiroa, four candidate genes PIF, MSI60, PERL1 and CALC1were not significantly different in the pearl sac between the two donor age categories. ASP had higher relative gene expression ratio in pearl sac from younger donor tissue (1.73).

Among the eight candidate genes studied in the pearl sac at Gambier, only the expression of SHEM5 was significantly different in the pearl sac between the two donor age categories (p = 0.0002). SHEM5 and ASP had higher relative gene expression ratio in pearl sac from younger donor tissue (4.68 and 1.5, respectively).

### Age effect on shell growth

**Fig 4:**
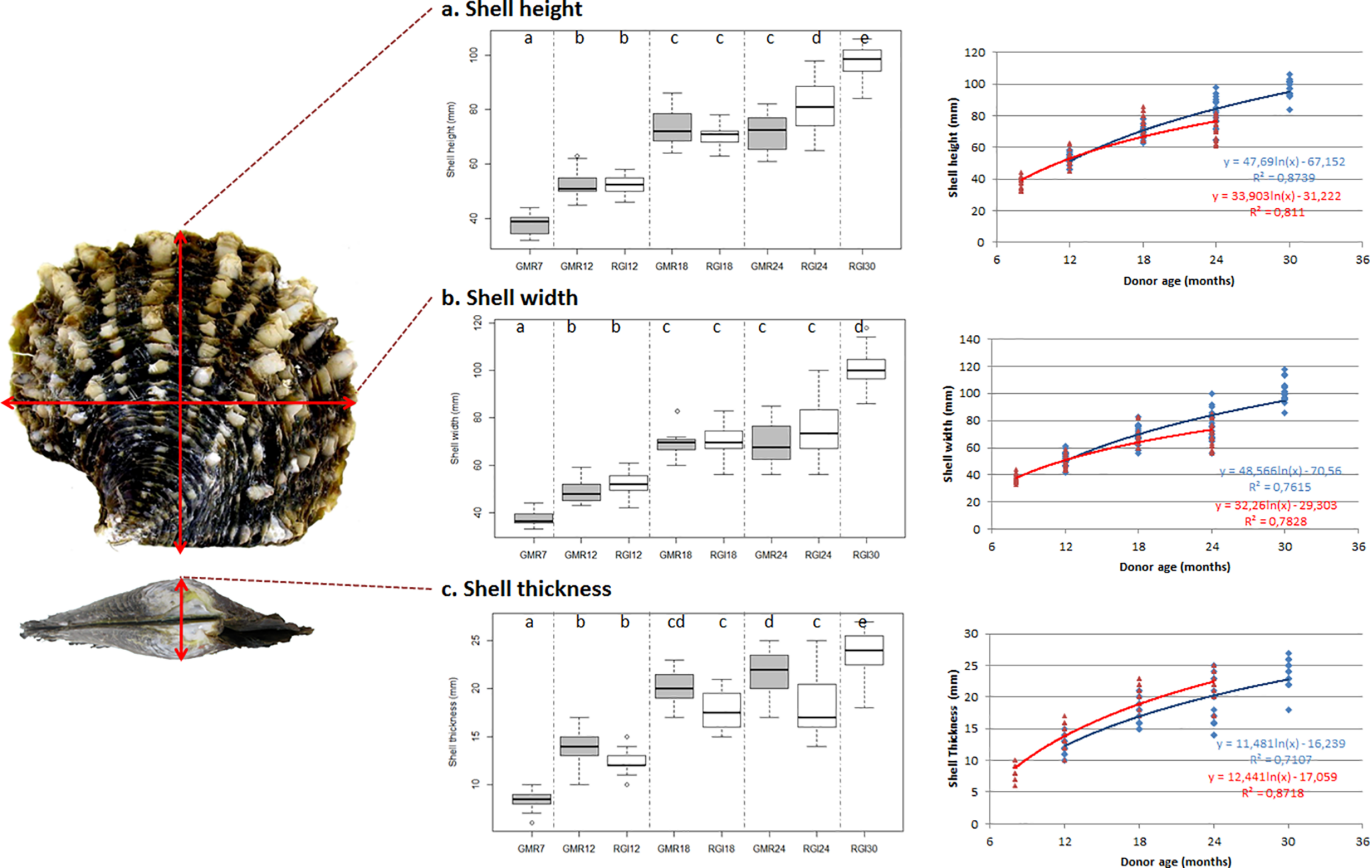
*Pinctada margaritifera* donor oyster shell biometric parameters. a. Shell height, b. width and c. thickness were measured at each graft time (GMR7 corresponds to 7 months old at Gambier location, GMR12 and RGI12 to 12 months old, GMR18 and RGI18 to 18 months old and GMR24 and RGI24 to 24 months and RGI30 to 30 months old at Rangiroa location). Each box-plot has the following elements: 1) median (solid bar in the box-plot); 2) 25^th^ to 75^th^ percentile (rectangular box); 3) 1.5*interquartile range (non-outlier range of the box whiskers); 4) minimum and maximum values (extreme dots); and 5) outlier values (outside box whiskers). Statistical differences between the age groups are indicated by letters (*p* < 0.05). The growth curve on the right contains two curves: Shell growth in Rangiroa in blue and in Gambier in red with their corresponding equations. Datas available on S2 Appendix.

At the time of grafting, the donor oyster width, height and thickness were recorded to provide average growth measurements for the two families. Results for shell growth with respect to donor age and culture site are described in Fig 4. No difference in growth rates between the two sites were observed in oysters younger than 24 months, *i*.*e*., only 24 month old donor oysters at (RGI24 and GMR24) were significantly different between Rangiroa and Gambier sites for shell height (with average measurements of 81.0 ± 9.2 mm and 71.9 ± 6.7 mm, respectively) and shell thickness (with average measurements of 18.4 ± 3.4 mm and 21.4 ± 2.6 mm, respectively). The growth curves show the maximum slope (*i*.*e*., maximum growth rate) for donors younger than 18 months old at both Rangiroa and Gambier.

## Discussion

This study is the first to evaluate the impact of pearl oyster donor age on pearl size (including nacre weight and thickness) and on the expression levels for a panel of eight genes involved in shell biomineralisation in *P*. *margaritifera*. This study is also the first to examine the impact of pearl donor oysters aged less than 2 years on pearl quality traits. We found that donor age impacts the size of the pearl, pearl grade and surface quality. Donor age also impacts the relative gene expression ratio of aragonite-related genes (*Pif-177*, *MSI60*, *Perline*), calcite-related genes (*Aspein*, *Shematrin5*, *Prismalin*,) as well as the gene implicated in both layers (*Nacrein*) for graft tissue. For pearl sac tissues only calcite-related genes (*Aspein*, *Shematrin5*, *Prismalin*, *Shematrin9*) were impacted by donor age.

The major result of this study is that donor age has an impact on pearl size. In fact we found that pearls from the oldest donors, 30 months in Rangiroa and 24 months in Gambier, yielded the lightest and the thinnest pearls. The ageing of the donor’s mantle cells clearly alter the quantity of nacreous deposit. Grafts originating from young donors produced bigger pearls than grafts produced by older donors (more than 24 months). This result may be related to the donor shell’s growth curve where maximum growth occurs before 18 months. As a general rule, growth rates are directly related to bivalve age. *P margaritifera* undergoes rapid shell growth until it reaches 18 months and then the rate slowly begins to decrease as it begins to invest more energy into reproduction [40]. Pouvreau et al. [41] also confirmed that growth differences between atolls became highly significant for 2-year-old pearl oysters and similarly, we observed that 24-month-old donors were significantly different in shell height and thickness between Rangiroa and Gambier (in Rangiroa, oysters had greater heights and in Gambier, oysters were thicker). The pearl itself is structurally identical to the nacreous layer of the shell consisting of calcium carbonate aragonite [42]. This physiological background could explain the ability of grafts originating from younger individuals to produce bigger pearls in association with cellular growth activity and this idea was further supported by the graft biomineralisation analysis which showed a higher level of expression for the aragonite candidate gene (PIF, MSI60 and PERL) in the younger donor oyster. In a previous study, we demonstrated that donor shell biometry at fixed age was not correlated with pearl size while the recipient shell biometry impacted pearl size [30]. Pearl size results from a complex interplay between the donor and the recipient oyster. When the donor is in a “growth period” (i.e. young stage), the cell’s graft will deposit more nacre on the pearl and if paired with a recipient oyster also undergoing a period of elevated growth, we would expect pearl size to be maximized. A previous study also lead to a similar hypothesis where they showed a relationship between shell growth performances of families selected and used as graft donors and the final weight of the cultured pearls produced [43]. In Pouvreau et al. [40], increments in nacre deposition equalled 7.2 μm.d^-1^ during the second year of the life cycle and decreased with the age of the pearl oyster, reaching a mean value of 3.1μm.d^-1^ during the fourth year of the life cycle and confirming that growth rate in shell or tissue is directly related to oyster age.

Age for donor and recipient oysters is a parameter that is important to consider. A donor aged between 12 and 18 months seems to be the ideal candidate for a maximum nacre deposition. In French Polynesia, donor oysters are currently sourcedfrom wild spat collection and used in operations after 24 months of age. Our results suggest that better results would be obtained by using younger oysters. The results were consistent with the graft biomineralisation analysis where in GMR30, the oldest donor oysters showed the lowest level of expression for the aragonite candidate genes (PIF, MSI60 and PERL). PIF was previously found to be positively correlated with shell deposition rates in *P*. *margaritifera* [35] and thus with pearl size (nacre weight and thickness) [27]. Among the various phenotypes of pearl quality traits surveyed in this work, those concerning grade and surface defects deserve special consideration. Indeed results concerning lustre are difficult to interpret. Lustre is known to depend on environmental factors such as temperature during cultivation period [25–26]. Our results clearly demonstrate that poorer pearls grade (more D-R pearls) and increased pearl defects (> 5)were observed when originated from donor oysters older than 24 months and younger than 12 months. For pearls produced by donors younger than 12 months, we propose a technical problem verses a physiological one. We hypothesise that a technical problem occurs because the use of very young oysters (i.e. small sized individuals, 3.78 ± 0.36 cm mean height) for the graft limits the precision in choosing the perfect tissues for the graft and leads to poor pearl quality. For pearls produced from donors older than 24 months, the aging of the pearl sac cells is likely to explain the reduction in pearl quality. This result is supported by an analysis of the expression level for the panel of 8 genes involved in the biomineralization of graft tissue. Results showed a minimum expression for genes involved in aragonite production in 24-month-old donor oysters from both Gambier and Rangiroa. Older cells are characterized by several detrimental changes that are likely to alter gene expression. We demonstrated in a recent study that the recipient oyster regulates the metabolism of the pearl sac by supplying nutrition throughout the pearl formation period [28,30,44]. Recipient oysters might regulate the expression of biomineralization genes in the pearl sac and this could explain the weak difference between donor age categories for pearl sac expression levels. We confirmed results reported by Ky et al. [31] where 2-year-old graft cells improved pearl grade, predominantly through a higher proportion of zero surface defects.

In the present study we can conclude that using donors that are too young (<12 months) or too old (>24 months) decreases the quality of the pearls produced (the current age in commercial production is > 24 months). This study demonstrates that donor age influences the pearl phenotype and that there is potential to improve pearl size and quality in *P*. *margaritifera* if donor age is optimized. Donor age (between 12 and 18 months) with high potential for nacre deposition and high biomineralization potential will increase cultured pearl size and quality.

## Supporting information

S1 Table Set of forward and reverse primers used for the biomineralization gene expression analysis in *Pinctada margaritifera*. (DOCX)

S1 Appendix Relative stability of the GAPDH and SAGE genes combination confirmed using NormFinder (Stability value for best combination). (DOCX)

S2 Appendix All quantitative and qualitative figures datas. (XLSX)
