# Supplementary material for: Optimal age of the donor graft tissue in relation to cultured pearl phenotypes in the mollusc, Pinctada margaritifera
Source: PLoS One. 2018 Jun 18;13(6):e0198505. doi: 10.1371/journal.pone.0198505 (PMC6005463; doi:10.1371/journal.pone.0198505)
Supplement: S1 Table — (DOCX) [file pone.0198505.s001.docx]

**Table S1 :** Set of forward and reverse primers used for the biomineralization gene expression analysis in *Pinctada margaritifera*.

| **Primer name** | **Protein name** | **Function** | **GenBank Accession Numbers** | **Forward primer (5’-3’)** | **Reverse primer (5’-3’)** | **Efficiency** |
| --- | --- | --- | --- | --- | --- | --- |
| **PIF** | Pif-177 | Aragonite formation | HE610401 | AGATTGAGGGCATAGCATGG | TGAGGCCGACTTTCTTGG | 2.02 |
| **MSI60** | MSI60 | Aragonite formation | No accession number but described by B. Marie et al.2012 | TCAAGAGCAATGGTGCTAGG | GCAGAGCCCTTCAATAGACC | 2.08 |
| **PERL1** | Perline | Aragonite formation | DQ665305 | TACCGGCTGTGTTGCTACTG | CACAGGGTGTAATATCTGGAACC | 2.04 |
| **ASP** | Aspein | Calcite formation | No accession number but described by B. Marie et al.2012 | TGGAGGTGGAGGTATCGTTC | ACACCTGATACCCTGCTTGG | 2.01 |
| **PRISM** | Prismalin 14 | Calcite formation | HE610393 | CCGATACTTCCCTATCTACAATCG | CCTCCATAACCGAAAATTGG | 1.92 |
| **SHEM5** | Shematrin | Calcite formation | HE610376 | GTCCGAAACCAAATCGTCTG | CTGTGGTGATGGTGACTTCG | 2.11 |
| **CALC1** | Nacrein | Aragonite and calcite formation | HQ896199 | CTCCATGCACAGACATGACC | GCCAGTAATACGGACCTTGG | 1.96 |
| **SHEM9** | Shematrin | Calcite formation | No accession number but described by B. Marie et al.2012 | TGGTGGCGTAAGTACAGGTG | GGAAACTAAGGCACGTCCAC | 1.95 |
