# Supplementary material for: Optimal age of the donor graft tissue in relation to cultured pearl phenotypes in the mollusc, Pinctada margaritifera
Source: PLoS One. 2018 Jun 18;13(6):e0198505. doi: 10.1371/journal.pone.0198505 (PMC6005463; doi:10.1371/journal.pone.0198505)
Supplement: S1 Appendix — (DOCX) [file pone.0198505.s002.docx]

**Appendix S1**: Relative stability of reference genes (GAPDH, SAGE and EF) used for RT-PCR analysis.

| Reference gene | Stability value |
| --- | --- |
| EF | 0.016 |
| **GAPDH** | **0.014** |
| SAGE | 0.023 |
| GAPDH and SAGE | 0.011 |
